# The Role of Paracellular Transport in the Intestinal Absorption and Biopharmaceutical Characterization of Minoxidil

**DOI:** 10.3390/pharmaceutics14071360

**Published:** 2022-06-27

**Authors:** Milica Markovic, Moran Zur, Sapir Garsiani, Daniel Porat, Sandra Cvijić, Gordon L. Amidon, Arik Dahan

**Affiliations:** 1 Department of Clinical Pharmacology, School of Pharmacy, Faculty of Health Sciences, Ben-Gurion University of the Negev, Beer-Sheva 8410501, Israel; milica@unc.edu (M.M.); moranfa@post.bgu.ac.il (M.Z.); sapirgar@post.bgu.ac.il (S.G.); poratdan@post.bgu.ac.il (D.P.); 2 Department of Pharmaceutical Technology and Cosmetology, Faculty of Pharmacy, University of Belgrade, Vojvode Stepe 450, 11221 Belgrade, Serbia; gsandra@pharmacy.bg.ac.rs; 3 Department of Pharmaceutical Sciences, College of Pharmacy, University of Michigan, Ann Arbor, MI 48109-1065, USA; glamidon@umich.edu

**Keywords:** Biopharmaceutics Classification System (BCS), drug permeability, intestinal absorption, minoxidil, paracellular drug transport, permeability pathways

## Abstract

The purpose of this study was to evaluate mechanisms behind the intestinal permeability of minoxidil, with special emphasis on paracellular transport, and elucidate the suitability of minoxidil to be a reference drug for Biopharmaceutics Classification System (BCS). The permeability of minoxidil (vs. metoprolol) was evaluated in-silico, in-vitro using both the PAMPA assay and across Caco-2 cell monolayers, as well as in-vivo in rats throughout the entire intestine. The permeability was studied in conditions that represent the different segments of the small intestine: upper jejunum (pH 6.5), mid small intestine (pH 7.0), distal ileum (pH 7.5), and colon (pH 6.5). Since we aimed to investigate the paracellular transport of minoxidil, we have also examined its permeability in the presence of quercetin (250 µM), which closes the tight junctions, and sodium decanoate (10 mM), which opens the tight junctions. While metoprolol demonstrated segmental-dependent rat and PAMPA permeability, with higher permeability in higher pH regions, the permeability of minoxidil was pH-independent. Minoxidil PAMPA permeability was significantly lower than its rat permeability, indicating a potential significant role of the paracellular route. In rat intestinal perfusion studies, and across Caco-2 monolayers, tight junction modifiers significantly affected minoxidil permeability; while the presence of quercetin caused decreased permeability, the presence of sodium decanoate caused an increase in minoxidil permeability. In accordance with these in-vitro and in-vivo results, in-silico simulations indicated that approximatelly 15% of minoxidil dose is absorbed paracellularly, mainly in the proximal parts of the intestine. The results of this study indicate that paracellular transport plays a significant role in the intestinal permeability of minoxidil following oral administration. Since this permeation route may lead to higher variability in comparison to transcellular, these findings diminish the suitability of minoxidil to serve as the low/high BSC permeability class benchmark.

## 1. Introduction

Biopharmaceutics Classification System (BCS) is a framework used in drug development to categorize all drugs into four different categories based on their aqueous solubility and permeability [1]; according to the BCS, drugs are divided into four categories: Class I (high solubility–high permeability), Class II (low solubility–high permeability), Class III (high solubility–low permeability), and Class IV (low solubility–low permeability) [1]. This classification aids the development of new compounds and provides regulatory guidelines that facilitate the development of optimal drug products. It was adopted by the United States Food and Drug Administration (FDA) and the European Medicine Agency (EMA) allowing a waiver for studies of clinical bioequivalence [2,3]. In addition, the BCS classification also allows a waiver for orally administered immediate release (IR) Class I drugs in solid dosage products [2], making the BCS biowaiver an important tool for saving cost/time/effort in the drug discovery/development process [4,5].

For a drug to be considered highly permeable, and classified into BCS class I or II, 90% of an orally administered drug needs to be absorbed [2]. Intestinal permeability (*P_eff_)* evaluation relies on defining the boundary for the low/high permeability class membership. If the permeability of an investigated drug is equal/higher than the reference compound with high permeability, the investigated drug can also be classified as highly permeable [2]. Until now, the FDA has listed few compounds as references for high permeability criterion [2]. One of them is metoprolol, chosen for its complete human intestinal absorption. A commonly used standard for high/low permeability classification is the permeability value for metoprolol in the jejunum [6,7,8]. Nevertheless, metoprolol is a conservative low/high permeability boundary with full absorption in human beings, due to urinary excretion of the unchanged drug and its total radioactive metabolites following oral and intravenous administration in healthy volunteers [9]. Additionally, it was shown that several drugs have complete absorption, despite having lower permeability than metoprolol. In such cases metoprolol can be viewed as a rigid example, which can lead to unsuitable classification (i.e., a drug can be classified as a low permeability drug, where in fact a drug merits high permeability classification).

One of the drugs that bears resemblance to metoprolol is minoxidil, both in terms of absorptive site (proximal jejunum) [10] and extensive metabolism, as well as urinary excretion (~10% of unchanged minoxidil was found in the urine) [11,12]; it was thus assumed that minoxidil might be a good candidate as a new high permeability boundary for BCS. Minoxidil is a potent vasodilator drug first approved in 1979 for severe hypertension management in patients who do not respond to conventional antihypertensive therapies [13,14,15]. Following the discovery of its common adverse event, hypertrichosis, minoxidil was developed as a topical formulation for promoting hair growth [16]. Nowadays oral formulations for this indication are investigated as well [17].

Following oral administration, a drug needs to cross the intestinal membrane through passive diffusion or active transport. Passive diffusion relies on two pathways: paracellular, where a drug diffuses between enterocytes through aqueous pores at the tight junctions; and transcellular, where a drug crosses through the enterocyte membrane. Physicochemical properties of each individual molecule guide their transport path through the luminal membrane [18]. Minoxidil (6-amino-1,2-dihydro-1-hydroxy-2-imino-4-piperidinopyrimidine) is a small model. The contribution of active vs. passive transport in the overall drug absorption process has been intensely studied in the past, whereas the contribution of transcellular vs. paracellular transport is much less investigated. It has been pointed out by our group in the past that minoxidil may be susceptible to paracellular transport [19], which could lead to variable permeability, contingent upon the state and condition of tight junctions in the gut (disease-, food effect-, physiology- and location-dependent variability) [20].

In this study, for the first time, we elucidated the contribution of paracellular vs. transcellular transport to the permeability of minoxidil. Using in-vitro, in-vivo and in-silico methods, we performed a thorough evaluation of the minoxidil intestinal permeability mechanisms, with special emphasis on paracellular transport. Minoxidil paracellular transport was investigated in the presence of tight junction modifiers, quercetin and sodium decanoate. Quercetin, a flavonoid compound, was shown to enhance intestinal tight junction barrier function through gathering and enhancing the expression of tight junction proteins [21,22]. Sodium decanoate (sodium caprate), on the other hand, opens the barriers of tight junction, allowing the molecules to pass through them more freely [23]. Considering the paracellular transport and novel findings presented hereinafter, an additional purpose of this work was to elucidate the suitability of minoxidil to be a BCS reference drug for low/high *P_eff_* class boundary standard.

## 2. Methods

### 2.1. Materials

Minoxidil, metoprolol, phenol red, sodium decanoate, quercetin, potassium chloride, potassium phosphate monobasic, potassium phosphate dibasic, sodium chloride, *n*-octanol, hexadecane, and trifluoroacetic acid (TFA) were all purchased from Sigma Chemical Co. (St. Louis, MO, USA). Acetonitrile and water, purchased from Merck KGaA, Darmstadt, Germany were of ultra-performance liquid chromatography (UPLC) grade. Other chemicals were of analytical reagent grade.

### 2.2. Octanol-Aqueous Buffer Partition Coefficient (Log D)

Minoxidil and metoprolol octanol-buffer partition coefficients, Log D, were experimentally determined at pH 6.5, 7.0, and 7.5 by the customary shake-flask method [24,25,26,27]. Drug solutions were prepared in octanol-saturated phosphate buffers with pH values of 6.5, 7.0, and 7.5. These aqueous solutions were equilibrated at room temperature (25 °C) with an equivalent volume of buffer saturated octanol for 48 h. Following centrifugation, water and octanol layer were separated, and the drug concentration in the water layer was determined via UPLC. The drug concentration in the octanol was determined by mass balance. From these data, the apparent octanol/buffer partition coefficient was calculated.

### 2.3. Physicochemical Analysis

The theoretical fraction extracted into octanol (*f_e_*) was calculated using the Equation (1) [28,29].

(1) $$f_{e}=\frac{f_{u}P}{1+f_{u}P}$$

where *P* presents the octanol-water partition coefficient of the unionized drug form and *f_u_* is the fraction unionized of the drug at a certain pH. Experimental literature Log P values were used for minoxidil [30] and metoprolol [31]. The *f_u_* vs. pH was plotted according to the Henderson–Hasselbalch equation, using the following literature pKa values: 9.68 for metoprolol [32] and 4.61 for minoxidil [33,34].

### 2.4. Parallel Artificial Membrane Permeability Assay (PAMPA)

Hexadecane-based PAMPA studies with minoxidil and metoprolol drug solutions were prepared with different ratios of potassium phosphate monobasic and sodium phosphate dibasic, to produce pH of 6.5, 7.0 and 7.5. Osmolality (290 mOsm/L) and ionic strength were maintained in all buffers. Millipore (Danvers, MA, USA) 96-well MultiScreen-Permeability filter plates with 0.3 cm^2^ polycarbonate filter support (0.45 µm) were impregnated with 15 µL of a 5% hexadecane in hexane solution and allowed to dry for 1 h, during which the hexane evaporated completely, producing a uniform layer of hexadecane. Following that, the donor wells were filled with the drug solution (200 µL), and the receiver wells were filled with a matched blank buffer (300 µL). The PAMPA sandwich was then incubated at room temperature. The receiver plates of both methods were collected at different time points. Apparent permeability coefficient (*P_app_*) values were calculated from the linear plot of the drug accumulated in the acceptor side vs. time using the equation:

(2) $$P_{app}=\frac{dQ/dT}{A*C_{0}}$$

where *dQ*/*dT* is the appearance rate of the drug on the receiver side, *C*_0_ is the initial drug concentration (80 µg/mL), and *A* is the membrane surface area (0.048 cm^2^).

### 2.5. Cell Culture and Caco-2 Permeability Measurements

Caco-2 cells that underwent passage 27–31 (American Type Culture Collection, Rockville, MD, USA) were routinely maintained in Dulbecco’s modified Eagle’s medium (Biological Industries USA, Cromwell, CT, USA) containing 10% fetal bovine serum, 1% nonessential amino acids, 1% sodium pyruvate, and 1% L-glutamine; cell cultures were cultivated in 5% CO_2_ and 90% relative humidity at 37 °C. Transport studies across Caco-2 cells were performed as per previously described protocol [35,36]. Caco-2 cells were seeded on semi-permeable filter inserts (6-well Transwell plate, Corning Costar Co., Cambridge, MA, USA) at ~150,000 cells per insert whose growth area was 4.7 cm^2^. The differentiation status of the monolayer was assessed by the transepithelial electrical resistance (TEER) instrument (Millicell-ERS epithelial Voltohmmeter, Millipore Co., Bedford, MA, USA). After 21 days, the TEER values of the monolayers reached the resistance values above 250–300 Ω cm^2^. On the day of the experiment, Caco-2 monolayers were rinsed and incubated with a blank buffer (20 min), which was then replaced with drug solutions. The first plate contained minoxidil solution (80 µg/mL, 382 µM)), the second plate contained minoxidil solutions with sodium decanoate (10, 5, 1 mM), and the third with quercetin (250, 100, 50 µM). Plates were kept on a shaking incubator (50 rpm, 37 °C) throughout the experiment. Samples (100 µL) were taken from the receiver side at various time points up to 120 min, and same volumes of blank buffer were added following the sample withdrawal. At the last time point (120 min) samples were also taken from the donor side as well, in order to confirm the mass balance. Apparent permeability coefficient (*P_app_*) values (cm/s) for the Caco-2 experiments were calculated from the linear plot of the drug transported to the acceptor side vs. time using the Equation (3):

(3) $$P_{app}=\frac{dQ/dT}{A*C_{0}}$$

where *dQ*/*dT* is the appearance rate of the drug on the receiver side, *C*_0_ is the initial drug concentration (80 µg/mL), and *A* is the membrane surface area (1.12 cm^2^).

### 2.6. Rat Single-Pass Intestinal Perfusion

The in-vivo effective permeability coefficient (*P_eff_*) of minoxidil vs. metoprolol in various intestinal segments was evaluated using the single-pass rat intestinal perfusion (SPIP) model. All animal experiments were performed according to the approved protocol by Ben-Gurion University of the Negev Animal Use and Care Committee (Protocol IL-60-11-2010). The animals (male Wistar rats weighing 230–260 g, Harlan, Israel) were housed and handled according to Ben-Gurion University of the Negev Unit for Laboratory Animal Medicine Guidelines. Prior to the experiment, the animals were fasted overnight (12–18 h) with free access to water. Rat allocation to different experimental groups was done at random. The intestinal permeability experiment followed previous reports [37,38,39,40,41]. In short, animals were anesthetized (intramuscular injection of 1 mL/kg ketamine-xylazine solution, 9%:1%, respectively, and positioned on a heated (37 °C) surface (Harvard Apparatus Inc., Holliston, MA, USA)). The abdomen was then exposed by a midline incision of 3–4 cm. The complexity of the entire small intestine (SI) was accounted for by measuring the effective permeability in three 10 cm segments: proximal segment of jejunum (beginning 2 cm below the ligament of Treitz), mid SI segment (isolated between the end of the upper and the beginning of the lower segments), distal segment of ileum (ending 2 cm above the cecum), and colon [42]. Each segment was cannulated on both ends and perfused with blank buffer. Working solutions contained minoxidil, metoprolol and phenol red (a non-absorbable marker for water flux measurements) and were prepared with potassium phosphate monobasic and sodium phosphate dibasic, to yield pH values of 6.5, 7.0 and 7.5, while maintaining similar osmolarity (290 mOsm/L) and ionic strength in all buffers throughout the experiment. In an additional experiment, metoprolol and minoxidil were perfused through mid SI segments together with sodium decanoate (10 µM) and quercetin (250 µM). Investigated therapeutic drug concentration was calculated by the highest dose strength/250 mL, where the highest single dose is 20 mg for minoxidil and 100 mg for metoprolol, and 250 mL is taken from bioequivalence (BE) study protocols that recommend administration of a drug product to fasting human volunteers with a glass ~8 oz of water. Drug concentrations in investigated solutions were minoxidil 80 µg/mL (382 µM) and metoprolol 40 µg/mL (1496 µM). All solutions were incubated in a 37 °C water bath. Steady-state environment was ensured by perfusing the drug-containing buffer for 1 h, followed by an additional 1 h of perfusion, during which sampling was done every 10 min. The pH of the collected samples was measured in the outlet sample, to verify that there was no pH change throughout the perfusion. All samples were assayed by UPLC. The length of each perfused intestinal segment was measured in the end of the experiment. The effective permeability (*P_eff_*; cm/s) through the rat gut wall was determined according to the Equation (4):

(4) $$P_{eff}=\frac{-Qln\;\left({C^{\prime }}_{out}/{C^{\prime }}_{in}\right)}{2\pi RL}$$

where *Q* presents the perfusion buffer flow rate (0.2 mL/min), ${C^{\prime }}_{out}/{C^{\prime }}_{in}$ is the ratio of the outlet/inlet drug concentration adjusted for water transport, *R* is the radius of the intestinal segment (conventionally used as 0.2 cm), and *L* is the length of the perfused SI segment [42,43,44].

### 2.7. Analytical Methods

Drug concentration was evaluated using an UPLC instrument Waters Acquity UPLC H-Class (Milford, MA, USA), containing a photodiode array detector and Empower software. Minoxidil and metoprolol were separated on Acquity UPLC XTerra C18 3.5 µm 4.6 × 250 mm column (Waters Co., Milford, MA, USA). Gradient mobile phase, going from 85:15% to 10:90% *v/v* 0.1% trifluoroacetic acid in water/acetonitrile, respectively, over 10 min on a flow rate of 1 mL/min (25 °C). The detection wavelength of minoxidil, metoprolol and phenol red were 280, 230, 423 nm, respectively. The inter- and intraday coefficients of variation were <1.0% and 0.5%, respectively. UPLC injection volumes for all analyses were 20 µL.

### 2.8. Statistical Analysis

Log D studies were replicated with n = 6, PAMPA and Caco-2 assays were n = 4. Animal studies were replicated with n = 5. All values are stated as means ± standard deviation (SD). Statistically significant differences between the experimental groups were evaluated by the nonparametric Kruskal–Wallis test for multiple comparisons, and the two-tailed nonparametric Mann–Whitney U test for two-group comparison. A *p* < 0.05 was considered statistically significant.

### 2.9. Physiologically-Based In-Silico Simulations

In-silico simulations were performed with the GastroPlus^TM^ software package (v. 9.7.0009, Simulations Plus Inc., Lancaster, CA, USA). The structure and functions of the software are described elsewhere [45,46]. In general, the software uses the Advanced Compartment Absorption and Transit (ACAT) model to simulate drug transit and absorption though nine consecutive segments of the gastrointestinal (GI) tract (stomach, duodenum, two jejunal segments, three ileal segments, caecum and ascending colon), and the processes a drug undergoes in the body over time are described by the set of differential equations. Fasted state physiology ACAT model of the human adult representative was used to simulate minoxidil oral absorption.

Drug-related input parameters were experimentally determined, taken from the literature or in-silico predicted, as depicted in Table 1 and Table 2. To elucidate in more detail, experimental data from rat perfusion studies were used to estimate minoxidil total permeability through distinct segments of the GI tract.

Segmental-dependent input values were selected based on experimental pH in rat studies and regional pH values of the human GI tract (Table 2). The conversion from rat to human values was performed using the software integrated permeability converter tool. Paracellular drug permeability in each intestinal compartment was calculated using the software default Adson model, based on drug properties (molecular radius (the software predicted from drug molecular weight), charge, diffusion coefficient) and physiological parameters for each compartment (pore radius, porosity, pore length). Transcellular permeability (passive diffusion) referred to the difference between total and paracellular drug permeabilities in each GI region.

To describe drug distribution and elimination following oral absorption, two compartmental pharmacokinetic model was used. The relevant pharmacokinetic parameters (clearance (CL), volume of distribution in central compartment (V_c_) and distribution constants k_12_ and k_21_) were optimized to best match the simulated minoxidil plasma profiles following oral drug administration with the mean profiles observed in the in-vivo study reported in the literature [10]. The optimized values, along with the calculated total volume of distribution (V_tot_) and elimination half-life (t_1/2_), were compared with the range of values reported in the literature.

The prediction power of the generated model was assessed by comparison of the simulated data (fraction of drug absorbed (F_abs_), maximum plasma concentration (C_max_), time to reach C_max_ (t_max_) and area under the plasma concentration-time curve (AUC_0-∞_) value) with the in-vivo data from the literature [10]. Graphical data from the literature were digitized using the DigIt™ program (version 1.0.4, Simulations Plus, Inc., Lancaster, CA, USA). The percent prediction errors (*PE*(%)) between the observed and predicted C_max_, t_max_ and AUC_0-∞_ values were calculated using the following equation:

(5) $$PE\left(\%\right)=\frac{\left(Observed\;value-Predicted\;value\right)\times 100}{Observed\;value}$$

## 3. Results

### 3.1. Octanol-Buffer Partition Coefficients

**Figure 1:**
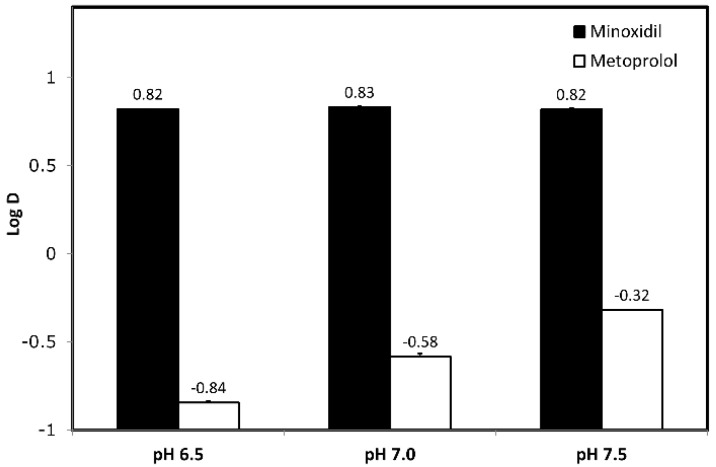
Log D values obtained for minoxidil (vs. metoprolol) at the three pH values 6.5, 7.0 and 7.5. Mean + SD; n = 6.

Minoxidil and metoprolol octanol-buffer partition coefficients (Log D) values were studied at pH values of 6.5, 7.0, and 7.5, indicative of the conditions present all through the intestine (Figure 1). Minoxidil did not demonstrate pH-dependent variations in octanol-buffer partition coefficient at different pH values. Metoprolol, on the other hand, has an upward increase in Log D values, respective of the pH-dependency from lower (6.5) to higher pH (7.5). Moreover, the octanol-buffer partition coefficient of minoxidil is higher than that of metoprolol at any given pH value.

### 3.2. Physicochemical Analysis

**Figure 2:**
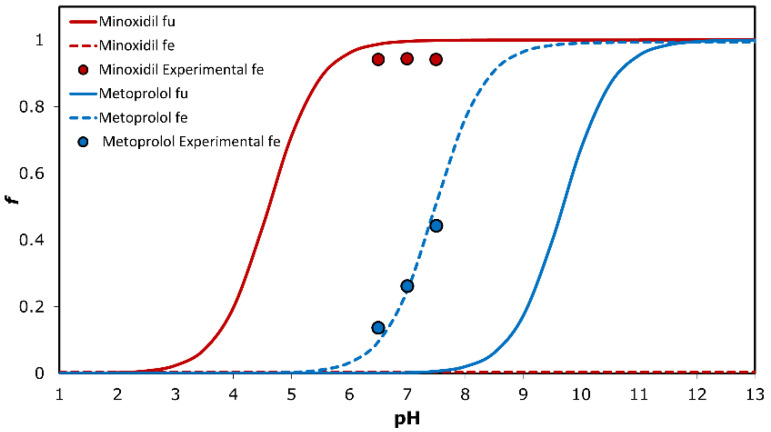
The theoretical fraction unionized (*f_u_*) and fraction extracted into octanol (*f_e_*) plots as a function of pH for minoxidil and metoprolol, and experimental buffer-octanol partitioning of the drugs in the three pH values 6.5, 7.0 and 7.5 (n = 6).

The theoretical fraction unionized (*f_u_*) and fraction extracted to the octanol (*f_e_*) plots are shown in Figure 2 as a function of pH for minoxidil vs. metoprolol. The *f_u_* of the basic drugs minoxidil and metoprolol is insignificant at low pH, and rises as the pH values increase, providing a standard sigmoidal shape. *f_e_* of minoxidil is negligible, whereas *f_e_* of metoprolol curves reach the same plateau, but it can be seen that the *f_e_* vs. pH plot for metoprolol shows a shift to the left at the lower pH values. The shift degree equals to Log (P − 1) at the midpoint of the *f_e_* and *f_u_* sigmoidal curves. Experimental Log D values from Figure 2 for both drugs at pH values of 6.5, 7.0, and 7.5 are presented as circles.

### 3.3. PAMPA Assay

**Figure 3:**
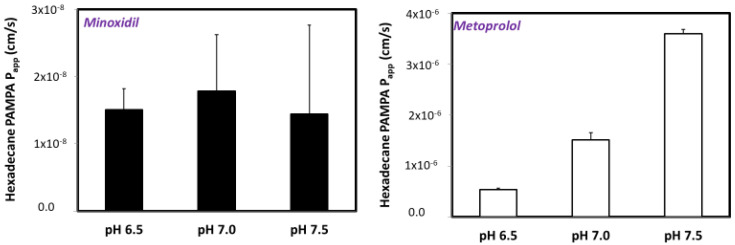
The apparent permeability (*P_app_*; cm/s) obtained for minoxidil and metoprolol at the three pH values 6.5, 7.0, and 7.5 in the hexadecane PAMPA experiments. Data are presented as the mean ± SD; n = 4 in each experimental group.

Hexadecane PAMPA transported amounts vs. time with their matching *P_app_* values for minoxidil and metoprolol are presented in Figure 3. PAMPA results were in good agreement with the Log D study. Minoxidil did not show a significant pH-dependent trend. In agreement with the Log D results, metoprolol showed the pH-dependent upward permeability trend. *P_app_* values for minoxidil were higher than values for metoprolol at lower pH values. However, at pH 7.5 metoprolol showed higher value than minoxidil, as can be seen in Figure 3.

### 3.4. Permeability Studies across Caco-2 Monolayer

**Figure 4:**
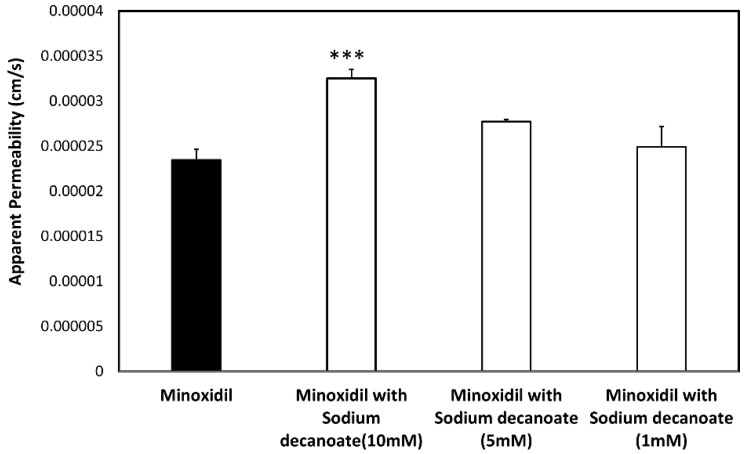
The permeability values of minoxidil alone and minoxidil with sodium decanoate in different concentration across Caco-2 cell monolayers. Mean ± SD; n = 4, *** *p*-value < 0.001.

**Figure 5:**
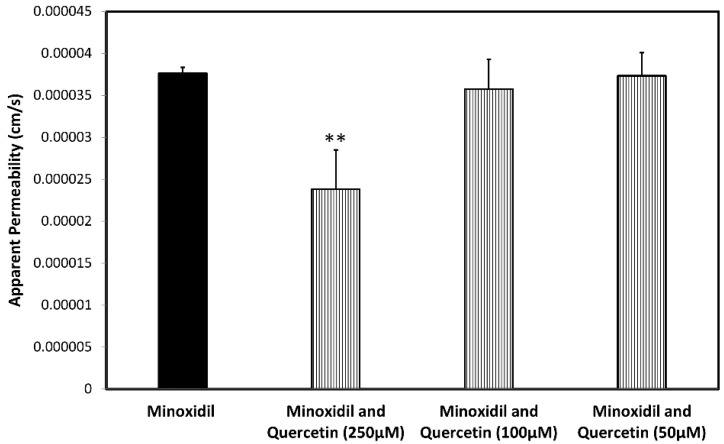
The permeability values of minoxidil alone and minoxidil with quercetin in different concentration across Caco-2 cell monolayers. Mean ± SD; n = 4, ** *p*-value < 0.01.

**Figure 6:**
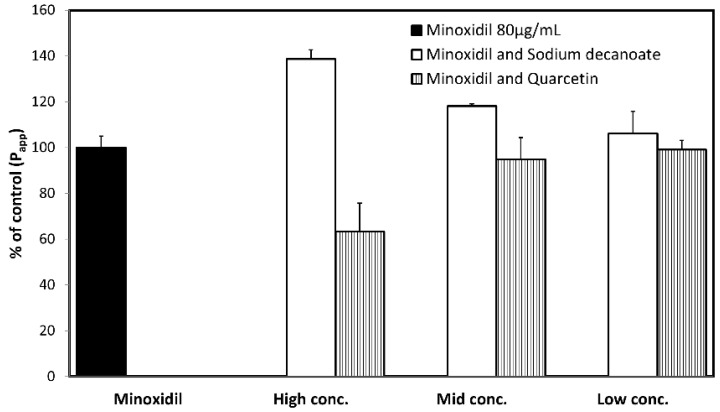
The percentage of control *P_app_* for minoxidil alone, and minoxidil with different concentrations of quercetin/sodium decanoate. Mean ± SD; n = 4.

The permeability values (*P_app_*; cm/s) of minoxidil with and without sodium decanoate (Figure 4) and quercetin (Figure 5) in the Caco-2 cell monolayers are presented in Figure 4 and Figure 5, respectively. Note that the tight junction modifiers (quercetin, sodium decanoate) significantly affected minoxidil’s permeability. Figure 6 presents the percentage of the control *P_app_* for minoxidil alone, and minoxidil with different concentrations of quercetin/sodium decanoate. While the presence of quercetin caused a decrease in minoxidil’s permeability, the presence of sodium decanoate lead to an increase in its permeability.

### 3.5. In-Vivo Rat Intestinal Perfusion (SPIP)

**Figure 7:**
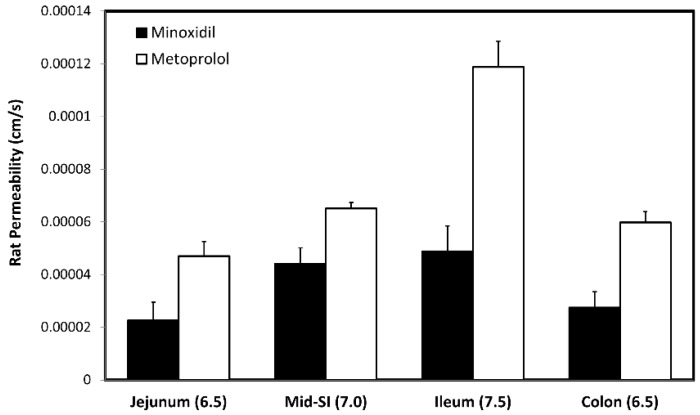
The effective permeability values (*P_eff_*; cm/s) obtained for minoxidil and metoprolol after in-situ single-pass perfusion to the rat proximal jejunum at pH 6.5, mid-small intestine at pH 7.0, distal ileum at pH 7.5, and to the colon at pH 6.5. Data are presented as the mean ± SD; n = 5 in each experimental group.

**Figure 8:**
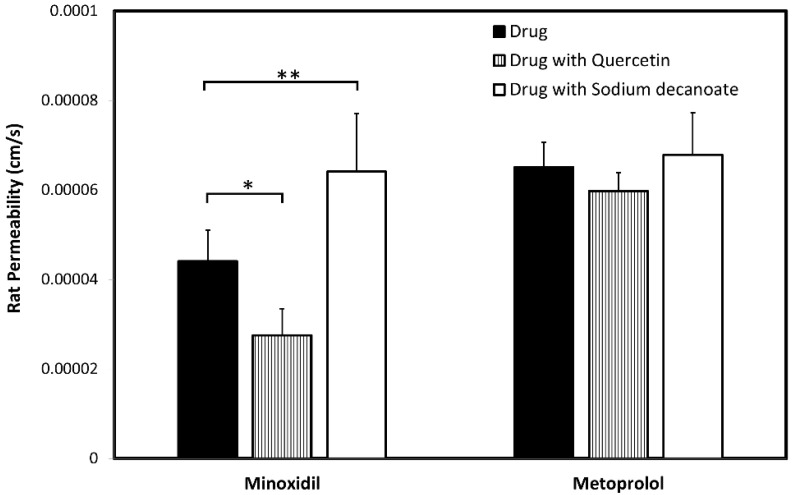
*P_eff_* obtained with and without the presence of tight-junction modifiers (quercetin/sodium decanoate). Data are presented as the Mean ± SD; n = 5 in each experimental group, * *p*-value < 0.05, ** *p*-value < 0.01.

The effective permeability coefficient (*P_eff_*, cm/s) values of minoxidil and metoprolol at therapeutic concentration, 80 µg/mL (382 µM) and 40 µg/mL (1496 µM), respectively, were determined using the single-pass intestinal perfusion (SPIP) rat model (Figure 7). Both drugs showed segmental-dependent intestinal permeability with higher permeability at higher pH (Figure 7). At any given intestinal segment/pH, the permeability of metoprolol was higher than that of minoxidil. To investigate the involvement of paracellular transport in the permeability of minoxidil, additional study was performed where the mid small intestinal segment was perfused with the tight junction modifiers quercetin and sodium decanoate (Figure 8). Indeed, in accordance with the Caco-2 results (Figure 4, Figure 5 and Figure 6), minoxidil permeability following concomitant intestinal perfusion with quercetin, an enhancer of tight junction barrier, led to a significant decrease in the permeability of minoxidil (Figure 8). In contrast, perfusion of minoxidil together with sodium decanoate, a tight junction opener, led to a significantly higher minoxidil *P_eff_* (Figure 8). No significant effect of tight junction modifiers was observed for metoprolol (Figure 8).

### 3.6. In-Silico Model Construction

**Figure 9:**
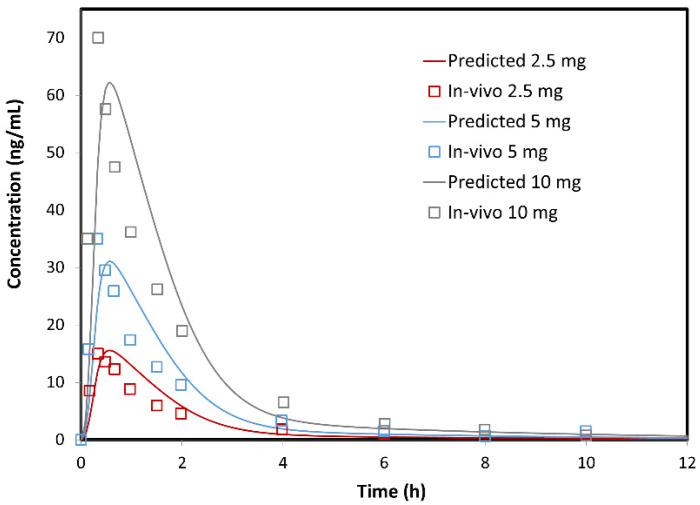
The simulated (lines) and mean in-vivo observed (markers) plasma concentration profiles following oral administration of different minoxidil doses. In-vivo data are taken from [10].

Minoxidil-specific in-silico model was constructed based on the input data provided in Table 1 and Table 2. To justify the selection of the input pharmacokinetic parameters, the optimized values were compared with the reported data. As shown in Table 3, all the data fit into the range of values reported in the literature. The resulting plasma concentration profiles for different oral drug doses are illustrated in Figure 9, together with the reference data from the in-vivo study. The predicted and observed pharmacokinetic parameters are given in Table 4. Figure 9 demonstrates that the in-silico profiles nicely match the mean profiles observed in the in-vivo study. Data from Table 4 also show that the predicted pharmacokinetic parameters fit the values from clinical studies.

Only the PE(%) for t_max_ are rather high, but it should be noted that these values were calculated in comparison to t_max_ from the mean plasma profiles. On the other hand, the predicted value of 0.56 h falls into the range of individual t_max_ (0.25–1.24 h). In addition, the predicted F_abs_ of cc. 99% is in agreement with the reported data of more than 90–95% drug absorbed [52,53]. Overall, the obtained results suggest that the generated model adequately describe minoxidil absorption and disposition following oral administration.

### 3.7. In-Silico Model Exploration

To elucidate minoxidil absorption pattern and assess contribution of paracellular and transcellular drug transport throughout the intestine, we have performed a more detailed analysis of the simulation outcomes. For the sake of clarity, only the generated data for 2.5 mg drug dose are discussed (the data for other tested drug doses coincide with these outcomes).

**Figure 10:**
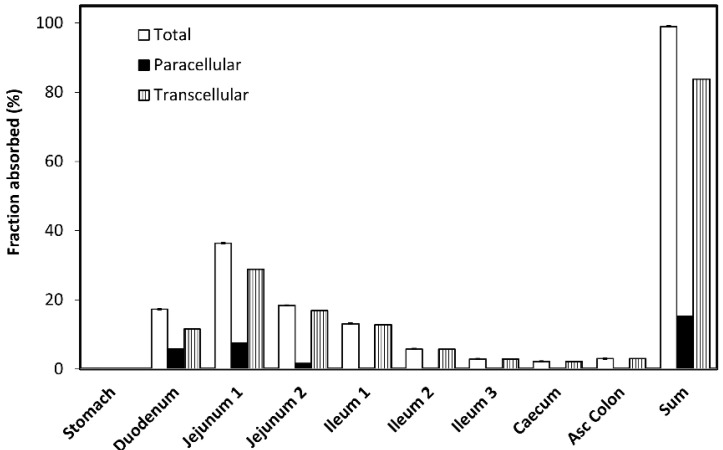
The simulated minoxidil absorption (total, paracellular and transcellular) from different regions in the human gastrointestinal (GI) tract.

The simulated minoxidil absorption pattern through different segments of the GI tract (Figure 10) reveals that the majority of drug is absorbed from the proximal intestine i.e., 72.1% from duodenum, jejunum 1 and jejunum 2. The rest of the dose is absorbed from mid GI regions, and some minor fraction from distal GI tract.

The predicted overall contribution of paracellular drug transport is 15.2%, while the remaining 83.8% of the drug is estimated to absorb via transcellular route (passive diffusion). Regional distribution of paracellular and passive transcellular minoxidil transport through the GI segments is also illustrated in Figure 10. According to the obtained data, paracellular transport of the drug predominantly happens in the proximal intestine (mostly in duodenum and jejunum 1) and decreased to zero in distal GI tract.

## 4. Discussion

For a drug to be the optimal BCS permeability class boundary, it needs to fulfill several criteria: (1) fraction dose absorbed ~90%; (2) absorption mainly through passive transcellular permeability, with no/insignificant carrier-mediated active intestinal transport; and (3) to have non-ionizable nature [27]. With respect to the F_abs_ of minoxidil and metoprolol, it has been demonstrated that they have complete absorption (at least 90% [52] and 100% [9], respectively). Luminal transport also plays a significant role in the permeability classification. Metoprolol and minoxidil were shown to be passively absorbed without involvement of carrier-mediated absorption [19]. However, with respect to minoxidil, studies suggest that while metoprolol has solely transcellular absorption, in minoxidil’s absorption the paracellular transport may play a significant role [19]. This may add considerable position dependence and variability in the permeability and consequent absorption of minoxidil.

Paracellular permeability is regulated by tight junction proteins (claudins, occludins) and is defined as the passage of water and/or small solutes through the spaces between neighboring epithelial cells [54,55]. Tight junctions between intestinal cells may vary in size depending on their position alongside villi, with greater openings at the base of the villi/crypts, and smaller openings at the tips of the villi. Recently it was shown that the permeability and composition of the intersections among Paneth, goblet, stem cells, and others colonizing the villus-crypt axis may differ largely [56,57]. Drugs that are absorbed via paracellular transport are mainly small (molecular weight < 250 Da), hydrophilic (Log P < 0) molecules. Minoxidil is indeed small molecule with molecular weight of 209.25 Da, with relatively low Log P (1.24) [58]. The complex of tight junctions has mainly negative net charge, hence molecules with positive charge are transported more easily than the negative ones, which are kept away. In the case of minoxidil, its formal charge is 0, making it a non-ionazable drug (in accordance to proposed properties for a boundary drug), which might be the reason for the absence of pH-dependent Log D and PAMPA results. In the Figure 1 and Figure 3, it is clear that while metoprolol has segmental/pH-dependent permeability, minoxidil is more pH-independent. For this reason, minoxidil could be a better low/high permeability boundary candidate than metoprolol. However, minoxidil’s small molecular weight suggests that paracellular transport through tight junctions may play a role in the intestinal permeability of the drug.

In light of these optimal characteristics, hereinafter, we aimed to investigate the permeability characteristics of minoxidil and compare them to metoprolol in order to assess its suitability as a reference drug for low/high permeability class boundary standard.

To evaluate the impact of paracellular transport on minoxidil permeability, we investigated the effect of tight junction modifiers. Indeed, Figure 4, Figure 5 and Figure 6 show that in the cell culture with tight junction modifiers, quercetin (closes the tight junctions) and sodium decanoate (opens the tight junctions), the absorption of metoprolol stays the same, while the absorption of minoxidil decreases/increases, respectively. These data are in excellent correlation with the rat SPIP perfusion studies (Figure 8), which clearly show the effect of tight junction modifiers on minoxidil’s permeability: higher permeability when minoxidil was perfused together with sodium decanoate, and lower permeability when administered with quercetin. In-silico simulation results coincide with these findings. Namely, the model estimated that about 15% of orally administered minoxidil is absorbed via paracellular route, mostly in proximal parts of the intestine. The obtained data can be explained by the estimated values for paracellular drug permeability in different GI regions, along with the physiological characteristics of the human GI tract (Table 5).

The largest portion of the drug passes through the aqueous pores in jejunum 1 due to relatively high paracellular drug permeability in this region and relatively large length and surface area of this intestinal segment. About one third of the paracellularly absorbed minoxidil passes through duodenum (the drug has the highest passive permeability in this region, but further passive absorption is limited by the length of duodenum). Some minor fraction of the drug is paracellularly absorbed in jejunum 2 and ileum 1, while the remaining GI regions do not contribute to this absorption route (the estimated paracellular permeabilities are negligible or zero).

It was shown that when predicting the absorption of partially paracellularly transported drugs there is significant variability in species used for absorption testing. It was shown on several drugs that the highest correlation is between human and rat, whereas testing in dogs resulted in overpredictive values [18]. Therefore, the single-pass intestinal perfusion method may elucidate the closest prediction to a human scenario for drugs that undergo paracellular transport. In human beings, tight junctions between the intestinal cells become tighter as we move from proximal (jejunum) to distal (colon) intestinal segments [18]. This explains the in-silico predicted zero paracellular permeability of minoxidil in the caecum and ascending colon. Namely, the pore radius in the caecum and ascending colon (3.92 A and 3.5 A, respectively) is smaller than the calculated minoxidil molecular radius of 3.99 A, hindering paracellular drug diffusion in these regions.

Altogether, in the context of an optimal BCS class permeability boundary, one may argue that the optimal *P_eff_* reference compound should show transcellular absorption, since paracellular absorption may add considerable position dependence and variability, which we want to avoid when we are choosing the low/high permeability boundary. This could also substantially interfere with the use of established in-vitro permeability systems such as cell culture method. All this contributed to our opinion that minoxidil may be an unsuitable low/high permeability marker. These findings may significantly contribute to better generic drug regulation, and help in various additional processes, such as absorption predictions and formulation development.

## 5. Conclusions

In this work, we evaluated biopharmaceutical properties of minoxidil and its suitability to be a BCS low/high permeability boundary. The combined in-silico/in-vitro/in-vivo work presented in this study demonstrates that paracellular transport plays a significant role in the intestinal permeability of minoxidil following oral administration. Since this permeation route may lead to higher variability vs. transcellular, these findings diminish the suitability of minoxidil to serve as the low/high BSC permeability class benchmark. In conclusion, this study, being one of the first on this topic, may serve as a framework for future detailed examination of paracellular drug transport, including both computational and experimental methods.

## Figures and Tables

**Table 1:** The selected input parameters for minoxidil absorption simulation.

Parameter	Value	Source
Molecular weight (g/mol)	209.25	/
Log D (pH 6.5)	0.82	experimental value
Solubility (aq) ^a^ (mg/mL)	2.2	[47]
pKa (base)	4.6	[48]
Human total permeability (cm/s)	see Table 2	/
Diffusion coefficient (cm^2^/s)	0.93 × 10^−5^	GastroPlus™ calculated value (based on molecular weight)
Mean precipitation time (s)	900	GastroPlus™ default values
Particle density (g/mL)	1.2	
Particle radius (µm)	25	
Blood/plasma concentration ratio	1.39	[49]
Plasma fraction unbound (%)	1	[50]
Clearance, CL (L/h/kg)	1.2	Optimized values
Volume of distribution in central compartment, Vc (L/kg)	0.1	
Distribution constant, k_12_ (1/h)	2.0	
Distribution constant, k_21_ (1/h)	0.2	
Volume of distribution in peripheral compartment, V_2_ (L/kg)	1.0	GastroPlus™ calculated
Elimination half-life, t_1/2_ (h)	4.05	
Dosage form	IR tablet	/
Dose (mg)	2.5; 5; 10	/

^a^ pH of minoxidil aqueous solution (pH 8.32) is software predicted, based on the drug pKa.

**Table 2:** Minoxidil permeability in relation to the regional pH values in the GI tract.

Experimental (Rat Studies)		Software Default Data	Predicted (Human) ^a^
Region/pH	Permeability (cm/s)	Region/pH	Permeability (cm/s)
Jejunum/pH 6.5	2.26 × 10^−5^	Duodenum/pH 6.0 Jejunum 1/pH 6.2 Jejunum 2/pH 6.4	1.27 × 10^−4^
Mid-SI/pH 7.0	4.41 × 10^−5^	Ileum 1/pH 6.6 Ileum 2/pH 6.9	2.22 × 10^−4^
Ileum/pH 7.5	4.89 × 10^−5^	Ileum 3/pH 7.4	2.43 × 10^−4^
Colon/pH 6.5	2.75 × 10^−5^	Caecum/pH 6.4 Asc Colon/pH 6.8	1.48 × 10^−4^

^a^ estimated using GastroPlus™ integrated permeability converter, based on experimental rat perfusion data.

**Table 3:** Comparison of the selected input values and literature data for minoxidil pharmacokinetic parameters.

Parameter	Optimized Input Value	In-Vivo Values (Range)	References
CL (L/h/kg)	1.2	0.5–1.5	[10,11,49,50,51]
V_tot_ (L/kg)	1.1	0.9–3.7	
t_1/2_ (h)	4.05	1.07–4.20 ^a^	

^a^ Commercial tablets label indicates the average minoxidil plasma half-life of 4.2 h [52].

**Table 4:** Comparison between the simulated and in-vivo observed minoxidil pharmacokinetic parameters following oral administration of different drug doses.

Parameter	In-Vivo Mean ^a^	Predicted	PE(%)
**2.5 mg dose**			
C_max_ (µg/mL)	15.00	15.55	−3.68
t_max_ (h)	0.34	0.56	−64.71
AUC_0→__∞_ (µg h/mL)	30.48	29.48	3.28
F_abs_ (%)	/	99.05	/
**5.0 mg dose**			
C_max_ (µg/mL)	35.00	31.12	11.09
t_max_ (h)	0.32	0.56	−75.00
AUC_0→__∞_ (µg h/mL)	61.46	58.99	4.02
F_abs_ (%)	/	99.09	/
**10 mg dose**			
C_max_ (µg/mL)	70.00	62.21	11.13
t_max_ (h)	0.34	0.56	−64.71
AUC_0→__∞_ (µg h/mL)	116.57	117.93	−1.17
F_abs_ (%)	/	99.05	/

^a^ Refer to the mean plasma profiles from [10].

**Table 5:** In-silico estimated segmental-dependent paracellular and transcellular minoxidil permeabilities, and relevant characteristics of the human GI tract.

Compartment	Drug Permeability			Physiological Characteristics		
	Total Permeability (cm/s)	Paracellular Permeability (cm/s)	Transcellular Permeability (cm/s)	Length (cm)	Radius (cm)	SEF ^a^
*Stomach*	0	0		28.29	9.67	1
*Duodenum*	1.27 × 10^−4^	5.82 × 10^−5^	6.87 × 10^−5^	14.13	1.53	4.23
*Jejunum 1*	1.27 × 10^−4^	3.55 × 10^−5^	9.14 × 10^−5^	58.40	1.45	3.95
*Jejunum 2*	1.27 × 10^−4^	1.41 × 10^−5^	11.00 × 10^−5^	58.40	1.29	3.49
*Ileum 1*	2.22 × 10^−4^	0.43 × 10^−5^	22.00 × 10^−5^	58.40	1.13	3.03
*Ileum 2*	2.22 × 10^−4^	0.09 × 10^−5^	22.00 × 10^−5^	58.40	0.98	2.57
*Ileum 3*	2.43 × 10^−4^	0	24.00 × 10^−5^	58.40	0.82	2.11
*Caecum*	1.48 × 10^−4^	0	15.00 × 10^−5^	13.19	3.38	1.79
*Asc Colon*	1.48 × 10^−4^	0	15.00 × 10^−5^	27.65	2.41	2.48

^a^ Surface area enhancement factor that scales the differences in drug absorption along the GI tract due to changes in the surface area-to-volume ratio (e.g., due to different densities of villi and microvilli in different intestinal segments).
